# Enhanced Photocatalytic Removal of Selected Pharmaceuticals from MBR-Treated Wastewater Using a g-C_3_N_4_/rGO Nanocomposite Under UV Irradiation

**DOI:** 10.3390/molecules31132346

**Published:** 2026-07-03

**Authors:** Klaudia Całus-Makowska, Renata Caban, Robert Zarzycki, Tomasz Kamizela, Marcin Dośpiał, Anna Grobelak

**Affiliations:** 1 Faculty of Infrastructure and Environment, Czestochowa University of Technology, 42-200 Częstochowa, Poland; robert.zarzycki@pcz.pl (R.Z.); tomasz.kamizela@pcz.pl (T.K.); anna.grobelak@pcz.pl (A.G.); 2 Faculty of Production Engineering and Materials Technology, Czestochowa University of Technology, 42-201 Czestochowa, Poland; renata.caban@pcz.pl (R.C.); marcin.dospial@pcz.pl (M.D.)

**Keywords:** emerging contaminants, advanced oxidation processes, photocatalysis, graphene-based materials, tertiary wastewater treatment

## Abstract

The presence of pharmaceuticals in treated wastewater has become an environmental concern due to their persistence, biological activity, and incomplete removal in conventional wastewater treatment systems. In this study, a g-C_3_N_4_/rGO nanocomposite was synthesized via thermal polycondensation of melamine in the presence of reduced graphene oxide and evaluated as a photocatalyst for the degradation of selected pharmaceuticals in membrane bioreactor (MBR)-treated wastewater. The obtained materials were characterized using Fourier-transform infrared spectroscopy (FTIR–ATR), X-ray diffraction (XRD), nitrogen adsorption–desorption measurements (BET), Raman spectroscopy, scanning electron microscopy (SEM), and UV–Vis spectroscopy to evaluate their chemical structure, crystallinity, textural properties, morphology, and optical characteristics. Photocatalytic experiments were performed under UV irradiation using real wastewater spiked with carbamazepine, diclofenac, ibuprofen, and sulfamethoxazole at an initial concentration of 50 mg/L, selected to ensure reliable quantification under laboratory conditions. The complete removal of diclofenac and sulfamethoxazole was achieved within 30 min of treatment, while the presence of the nanocomposite enhanced the degradation efficiency of ibuprofen and carbamazepine by approximately 19% and 13%, respectively, compared to UV irradiation alone. The obtained results demonstrate the applicability of the investigated g-C_3_N_4_/rGO system for pharmaceutical degradation in real wastewater matrices and indicate its potential as a preliminary photocatalytic post-treatment approach.

## 1. Introduction

The continuous increase in the consumption of medicinal products associated with population growth, aging societies, and the widespread use of pharmaceuticals in human and veterinary medicine has resulted in the persistent release of biologically active compounds into aquatic environments [1]. Pharmaceutical residues, commonly classified as emerging contaminants, are frequently detected in municipal wastewater effluents due to their incomplete removal during conventional treatment processes [2]. Wastewater treatment plants are not specifically designed to eliminate micropollutants such as active pharmaceutical ingredients (APIs), and therefore many compounds may persist in treated effluents discharged into receiving waters [3]. Numerous studies have reported the occurrence of analgesics, antibiotics, anti-inflammatory drugs, and antiepileptic compounds in surface waters, groundwater, and wastewater effluents worldwide [4,5,6]. The environmental presence of these contaminants is associated with chronic toxicity toward aquatic organisms, endocrine disruption, bioaccumulation, and the promotion of antimicrobial resistance [6,7,8].

Among the pharmaceuticals commonly detected in wastewater systems, carbamazepine (CBZ) is considered one of the most recalcitrant compounds because of its high chemical stability and resistance to biodegradation [9]. Consequently, CBZ is frequently used as an indicator compound for evaluating the effectiveness of advanced wastewater treatment technologies. In contrast, diclofenac (DCF) and sulfamethoxazole (SMX) have attracted particular environmental attention due to their ecotoxicological effects and widespread occurrence in aquatic systems [10]. DCF has been identified as a priority pollutant because of its adverse impact on aquatic organisms, while SMX is regarded as environmentally relevant owing to its contribution to the development of antibiotic resistance. The behavior of sulfamethoxazole (SMX) in purification processes is strongly dependent on pH, as the compound can exist in various ionic forms, which influences both its sorption and reactivity during degradation processes [11]. Ibuprofen (IBU) is also frequently detected in municipal wastewater due to its extensive consumption and incomplete elimination during biological treatment. Studies on nitrifying activated sludge have shown biodegradation of IBU of up to 99%, with the rate of the process depending on the operating conditions of the reactor [12]. IBU is an acidic compound and can exist in either a non-ionized or ionized form. At environmental pH, typically higher than its pKa, the anionic form predominates, which affects its mobility in the aqueous phase and limits its simple removal by sedimentation. At the same time, the presence of a hydrophobic aromatic skeleton enables ibuprofen sorption onto carbon-based sorbents such as activated carbon, biochar, or composite materials [13]. Membrane bioreactors (MBRs) are considered among the most effective municipal wastewater treatment technologies due to their excellent biomass retention and high effluent quality [14,15]. The MBR is an advanced wastewater treatment technology that combines biodegradation with membrane separation, which allows maintaining a high biomass concentration and may improve the elimination of some pharmaceuticals compared to classic activated sludge [16]. Nevertheless, even advanced MBR systems are not specifically designed for the complete elimination of pharmaceutical micropollutants, resulting in the persistence of biologically active compounds in treated effluents and highlighting the need for effective post-treatment technologies [3].

Advanced oxidation processes (AOPs) have emerged as promising technologies for the degradation of persistent organic contaminants due to their ability to generate highly reactive oxygen species (ROS), including hydroxyl radicals (•OH), superoxide radicals (•O_2_^−^), and photogenerated holes (h^+^) [17,18,19,20]. Among AOPs, UV-based systems and combined UV/O_3_ processes are widely investigated because UV irradiation promotes the formation of reactive radicals responsible for contaminant degradation. In particular, UV/O_3_ systems have demonstrated high efficiency toward pharmaceutical removal due to enhanced ozone decomposition and intensified ROS generation [21]. However, despite their effectiveness, these technologies are associated with several limitations, including high energy demand, operational costs, and the necessity for ozone generation systems.

Photocatalysis based on semiconductor materials has attracted considerable attention as a sustainable and potentially energy-efficient approach for wastewater treatment [22,23,24]. Under light irradiation, semiconductor photocatalysts generate electron–hole pairs that initiate the formation of reactive oxygen species responsible for contaminant degradation. TiO_2_ is a classic photocatalyst, but it has limited activity under visible light and rapid charge carrier recombination. Therefore, it is often combined with other semiconductors or carbon materials. Moreover, carbon dot TiO_2_ composites are being developed specifically for photocatalytic removal of pharmaceuticals, as CQDs can act as photosensitizers, electron transport mediators, and light absorption enhancers [22,25]. Among the photocatalytic materials investigated to date, graphitic carbon nitride (g-C_3_N_4_) has emerged as a promising metal-free photocatalyst due to its suitable band gap, chemical and thermal stability, relatively low toxicity, and simple synthesis from nitrogen-rich precursors such as melamine [26,27,28]. In addition, g-C_3_N_4_ exhibits photocatalytic activity under both UV and visible-light irradiation, making it attractive for environmental applications. Nevertheless, pristine g-C_3_N_4_ suffers from several important limitations, including rapid recombination of photogenerated electron–hole pairs, relatively low specific surface area, and insufficient charge transport capability, which substantially decrease photocatalytic efficiency [28].

To overcome these limitations, numerous modification strategies involving carbon-based materials have been proposed, particularly the incorporation of reduced graphene oxide (rGO) into g-C_3_N_4_ structures [29,30,31]. rGO possesses excellent electrical conductivity and favorable electron transport properties, which may contribute to improved charge separation and reduced electron–hole recombination within the photocatalytic system. Moreover, the incorporation of rGO may enhance the formation and transfer of reactive oxygen species, including •OH, •O_2_^−^, and h^+^, thereby improving photocatalytic activity in complex wastewater matrices. The addition of graphene-based materials may also increase adsorption capacity and facilitate interfacial electron transfer between catalyst components. However, the photocatalytic performance of g-C_3_N_4_/rGO systems strongly depends on the physicochemical properties and reduction degree of graphene-based materials. Insufficient reduction of graphene oxide may negatively affect electron mobility and limit photocatalytic efficiency, highlighting the critical importance of synthesis conditions and material quality.

While numerous studies have investigated g-C_3_N_4_-based photocatalysts for environmental applications, most experiments have been performed using model aqueous solutions under simplified laboratory conditions and relatively low contaminant concentrations [29]. In contrast to ultrapure or deionized water systems, real wastewater matrices contain dissolved organic matter, inorganic ions, and competing constituents that may significantly influence photocatalytic processes through radical scavenging and competitive adsorption phenomena. Furthermore, limited research has evaluated the applicability of g-C_3_N_4_/rGO nanocomposites in membrane bioreactor (MBR)-treated wastewater under highly loaded pharmaceutical contamination. The use of real wastewater matrices is particularly important for assessing the practical applicability of photocatalytic systems under realistic environmental conditions [32].

Although g-C_3_N_4_-based photocatalysts are frequently investigated under visible-light irradiation, UV irradiation was selected in this study to ensure direct comparison with conventional UV-based wastewater treatment systems and the UV/O_3_ process commonly applied as tertiary treatment technologies in wastewater treatment plants. Therefore, aligning with the growing need for sustainable post-treatment strategies, the primary objective of this study was to evaluate the practical applicability of a g-C_3_N_4_/rGO nanocomposite for the removal of selected pharmaceuticals from real MBR-treated wastewater. To evaluate the physicochemical properties of the photocatalysts, both pristine g-C_3_N_4_ and the synthesized g-C_3_N_4_/rGO nanocomposite were characterized using Fourier-transform infrared spectroscopy (FTIR–ATR), X-ray diffraction (XRD), nitrogen adsorption–desorption analysis (BET), Raman spectroscopy, scanning electron microscopy (SEM), and UV–Vis spectroscopy. Subsequently, the photocatalytic activity of the system was extensively investigated under realistic, highly loaded conditions. The removal efficiencies of DFC, IBU, SMX, and CBZ were evaluated, and the performance of the UV/g-C_3_N_4_/rGO system was compared with the UV/O_3_ process to determine its potential applicability for the removal of hazardous micropollutants from biologically treated effluents.

## 2. Results and Discussion

### 2.1. Physicochemical Characteristics of MBR-Treated Wastewater

The physicochemical characteristics of the MBR-treated wastewater used in the experiments are presented in Table 1. The analyzed effluent exhibited relatively low concentrations of organic matter and nutrients, confirming the high efficiency of the applied membrane bioreactor system in the removal of conventional wastewater contaminants. In particular, the low BOD_5_ and COD values indicated effective biological degradation of biodegradable organic compounds prior to the advanced treatment stage. Similar observations have been widely reported for MBR systems, which are recognized for their high treatment performance and ability to produce high-quality effluents suitable for advanced polishing processes [3,14,33].

The treated wastewater was characterized by neutral pH, low ammonium concentration, and relatively low nutrient content, suggesting efficient nitrification and stable biological treatment conditions. Moreover, the obtained COD and BOD_5_ values were significantly below the typical discharge limits established for treated municipal wastewater effluents, further confirming the effectiveness of the MBR process. These findings are consistent with previous studies demonstrating that MBR systems may achieve removal efficiencies exceeding 90% for conventional pollutants while producing effluents with substantially lower suspended solids and organic load compared to conventional activated sludge systems [34].

**Table 1:** Physicochemical characteristics of the MBR-treated wastewater used in the photocatalytic experiments compared with typical discharge limits for treated municipal wastewater effluents.

Parameter	Unit	Measured Value	Discharge Limits *
pH	–	7.06–7.08	6.5–9.0
Dissolved oxygen (DO)	mg·L^−1^	10.61–10.81	n.r.
Chemical oxygen demand (COD)	mg O_2_·L^−1^	38.20–39.13	≤125
Biochemical oxygen demand (BOD_5_)	mg O_2_·L^−1^	5.0–6.0	≤25
Total nitrogen (N_tot_)	mg·L^−1^	8.98–9.13	≤10–15
Ammonium nitrogen (N–NH_4_^+^)	mg·L^−1^	0.24–0.27	≤10
Nitrate nitrogen (N–NO_3_^−^)	mg·L^−1^	3.06–3.31	n.r.
Nitrite nitrogen (N–NO_2_^−^)	mg·L^−1^	0.076–0.088	n.r.
Phosphate phosphorus (P–PO_4_^3−^)	mg·L^−1^	0.20–0.22	≤1–2
Chlorides (Cl^−^)	mg·L^−1^	143–145	n.r.

* Discharge limits refer to maximum allowable concentrations for treated municipal wastewater discharged into surface waters or soil, as specified in the Regulation of the Minister of Maritime Economy and Inland Navigation [35], applicable to wastewater treatment plants serving agglomerations of ≥10,000 PE. Parameters marked as n.r. (not regulated) are not subject to numerical limit values under the aforementioned regulation.

Despite the high treatment efficiency, the effluent still represented a complex and realistic wastewater matrix containing residual dissolved organic matter and inorganic ions that could potentially influence photocatalytic degradation processes. Such matrix constituents may act as radical scavengers, compete with target pharmaceuticals for reactive oxygen species, or partially attenuate UV irradiation, thereby affecting degradation kinetics. Similar effects have been previously described for advanced oxidation processes applied in real wastewater matrices, where the presence of background organic matter may significantly reduce the availability of hydroxyl radicals and other reactive oxygen species for target contaminant degradation [36].

Importantly, conducting experiments in real MBR-treated wastewater rather than in ultrapure or synthetic solutions substantially increases the environmental relevance and practical applicability of the obtained results. Although model aqueous systems are frequently used in photocatalytic studies, they often fail to reflect the complexity of real effluents and may therefore overestimate degradation efficiencies under environmental conditions.

### 2.2. Structural and Optical Characterization of g-C_3_N_4_/rGO Nanocomposite

#### 2.2.1. Analysis of FTIR-ATR Results

**Figure 1:**
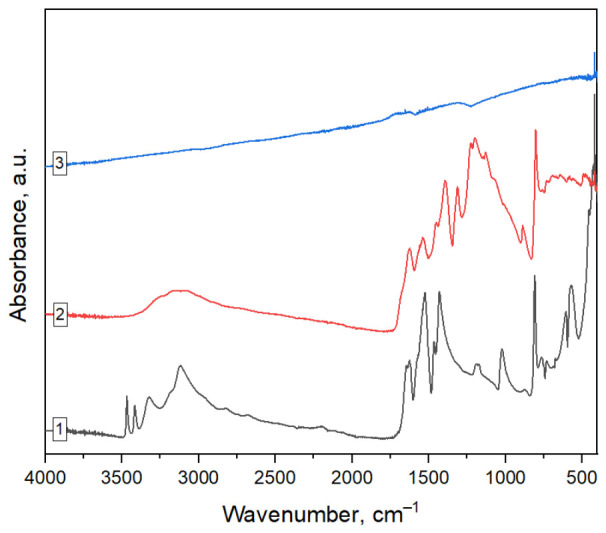
FTIR–ATR spectra of the investigated materials: 1—melamine, 2—g-C_3_N_4_/rGO nanocomposite, 3—reduced graphene oxide.

Comparison of the FTIR–ATR spectra (Figure 1) of the investigated materials reveals significant differences in band intensity and position, particularly in the wavenumber ranges of 3480–3100 cm^−1^ and 1650–1000 cm^−1^. The spectrum of melamine (curve 1) exhibits characteristic bands associated with the triazine ring structure and amino groups [37].

The peaks appearing in the wavenumber range of 3480–3100 cm^−1^ are attributed to the stretching vibrations ν(N–H) of amino groups (–NH_2_). The peaks observed in the range of 1650–1620 cm^−1^ are assigned to the deformation vibrations δ(N–H) and skeletal vibrations of the triazine ring. Peaks in the range of approximately 1550–1500 cm^−1^ correspond to C=N ring stretching and N–H bending vibrations. Peaks observed in the range of approximately 1460–1350 cm^−1^ are associated with C–N stretching vibrations ν(C–N).

The broadening of the band in the 3480–3100 cm^−1^ region and the changes observed around 1630 cm^−1^ in the spectrum of the nanocomposite (curve 2) indicate strong intermolecular interactions between the amino groups of melamine and the oxygen-containing functional groups of rGO, primarily through hydrogen bonding and possible partial formation of amide bonds, while the triazine ring structure is retained [29,38].

The band observed at approximately 810 cm^−1^ in the FTIR–ATR spectra is attributed to the characteristic out-of-plane bending vibrations of nitrogen-containing carbon rings, which constitute the fundamental structural unit of g-C_3_N_4_-type materials [39]. In the case of melamine, this band corresponds to the symmetric vibrations of a single triazine ring (C_3_N_3_), which, owing to the high symmetry of the system, exhibits a relatively narrow and well-defined profile. Upon thermal condensation of melamine, however, more complex heptazine units (C_6_N_7_) are formed, consisting of three fused triazine rings. In such a structure, the vibrations become coupled between adjacent rings, leading to a slight shift in the band towards lower wavenumbers (approximately 800 cm^−1^) and its broadening. The presence of this band, together with the intense signals in the 1650–1000 cm^−1^ range corresponding to C–N and C=N bond vibrations, provides significant confirmation of the successful formation of the g-C_3_N_4_/rGO nanocomposite.

#### 2.2.2. Analysis of X-Ray Diffraction Results

**Figure 2:**
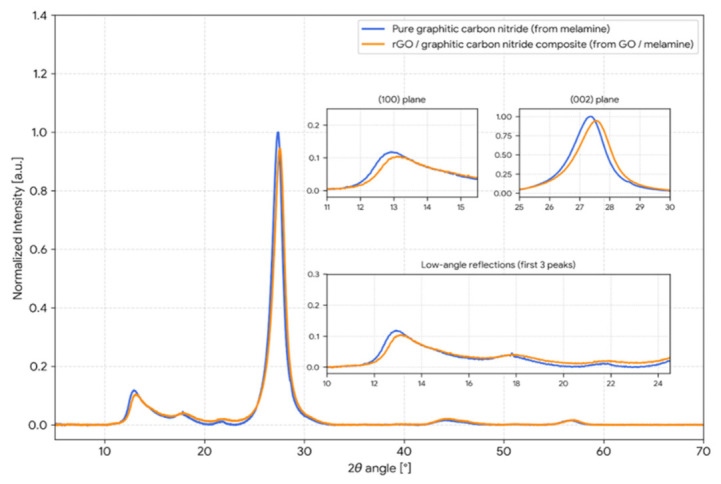
XRD patterns of pristine g-C_3_N_4_ and the g-C_3_N_4_/rGO nanocomposite. Insets present enlarged views of the characteristic diffraction peaks assigned to the (100) and (002) planes and the low-angle diffraction region.

The XRD pattern of pristine g-C_3_N_4_ and g-C_3_N_4_/rGO material is presented in Figure 2. Both materials exhibited the characteristic diffraction features of graphitic carbon nitride. The diffraction peak located at approximately 13.1° can be assigned to the (100) plane, corresponding to the in-plane structural ordering of tri-s-triazine units, whereas the intense reflection at approximately 27.4–27.6° corresponds to the (002) plane associated with the interlayer stacking of conjugated aromatic layers in g-C_3_N_4_.

The XRD pattern of the g-C_3_N_4_/rGO nanocomposite retained the characteristic reflections of g-C_3_N_4_, indicating that the incorporation of rGO did not alter the fundamental crystalline structure of graphitic carbon nitride. However, slight differences in the intensity and shape of the (100) and (002) reflections were observed for the composite material. In particular, a small decrease in peak intensity together with slight peak broadening can be noticed, suggesting structural interactions between g-C_3_N_4_ and rGO sheets and a partial modification of the stacking arrangement of g-C_3_N_4_ layers.

No distinct diffraction peak characteristic of graphene oxide, typically reported near 10–11°, was observed in the composite sample. This observation suggests effective reduction in GO during thermal treatment and is consistent with the formation of reduced graphene oxide within the composite structure.

The overall similarity of the diffraction patterns confirms that the synthesis procedure preserved the graphitic carbon nitride framework while enabling the incorporation of rGO into the composite material.

#### 2.2.3. Analysis of Surface Area and Porosity (BET) Results

Table 2 summarizes the textural parameters of pristine g-C_3_N_4_ and the synthesized g-C_3_N_4_/rGO nanocomposite determined from N_2_ adsorption–desorption measurements, including the BET specific surface area, micropore characteristics, and BJH pore volume. The corresponding N_2_ adsorption–desorption isotherms and pore size distribution curves are provided in the Supplementary Materials (Figures S1 and S2, respectively). Both materials exhibited type IV adsorption–desorption isotherms with a relatively narrow hysteresis loop, characteristic of mesoporous structures. The overall shape of the isotherms remained similar after rGO incorporation, indicating preservation of the mesoporous framework, while subtle differences in the adsorption branch and pore size distribution reflected modifications in the pore architecture and the development of additional accessible porosity within the composite material.

Nitrogen adsorption–desorption measurements revealed moderate but distinct differences in the textural properties of pristine g-C_3_N_4_ and the g-C_3_N_4_/rGO nanocomposite (Table 2). The BET specific surface area increased from 10.17 ± 0.03 m^2^/g for pristine g-C_3_N_4_ to 11.49 ± 0.04 m^2^/g following rGO incorporation, corresponding to an increase of approximately 13%. Although the overall change in surface area was relatively limited, more pronounced differences were observed in the pore structure of the materials.

Pristine g-C_3_N_4_ did not exhibit measurable microporosity according to the t-plot analysis. In contrast, the g-C_3_N_4_/rGO nanocomposite displayed detectable micropores, with a micropore volume of 0.0033 cm^3^/g and a median micropore width of 1.16 nm. Furthermore, the BJH pore volume increased from 0.0637 cm^3^/g for pristine g-C_3_N_4_ to 0.0794 cm^3^/g for the composite, indicating the development of additional accessible porosity after rGO incorporation.

The observed increase in pore volume and the appearance of micropores suggest that the incorporation of rGO altered the packing arrangement of g-C_3_N_4_ layers and partially prevented their dense restacking during thermal synthesis. Such structural modifications are consistent with the SEM observations, which revealed a more corrugated and interconnected morphology for the composite material. The resulting porous architecture may facilitate the diffusion of pharmaceutical molecules toward photocatalytically active sites and improve mass-transfer processes during photocatalytic treatment.

Although the increase in BET surface area was relatively modest, the combined increase in pore volume and emergence of microporosity indicated that rGO contributed to the development of a more accessible surface structure. These textural changes may partly explain the enhanced photocatalytic performance of the g-C_3_N_4_/rGO nanocomposite compared with pristine g-C_3_N_4_ observed during pharmaceutical degradation experiments.

#### 2.2.4. Analysis of Raman Spectroscopy Results

**Figure 3:**
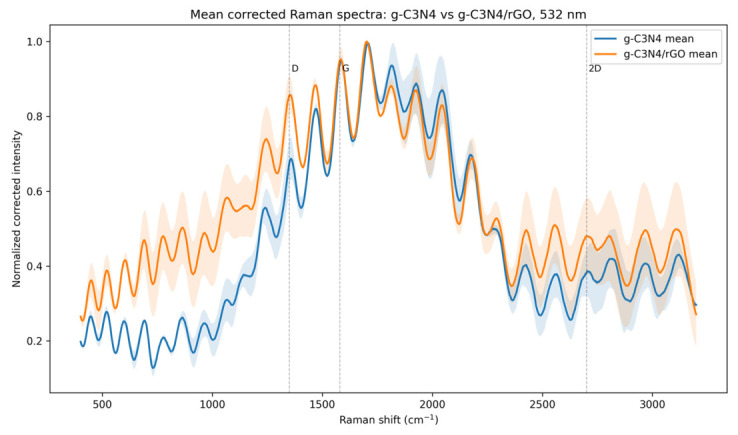
Raman spectra of pristine g-C_3_N_4_ and the g-C_3_N_4_/rGO nanocomposite recorded using a 532 nm excitation laser.

Raman spectroscopy provided complementary information on the structural properties of pristine g-C_3_N_4_ and the g-C_3_N_4_/rGO nanocomposite (Figure 3). The mean baseline-corrected and normalized spectra, obtained from two independent measurement points for each material, revealed clear differences after rGO incorporation. The Raman response of pristine g-C_3_N_4_ was dominated by bands associated with the vibrational modes of the conjugated heptazine-based framework, including C–N and C=N bonds. In comparison, the g-C_3_N_4_/rGO composite exhibited enhanced spectral intensity in several regions, indicating the contribution of additional carbonaceous structures introduced by reduced graphene oxide.

As shown in Figure 3, the most pronounced differences were observed in the regions corresponding to the characteristic D and G bands of graphitic carbon materials. Broad features centered at approximately 1355 cm^−1^ and 1582 cm^−1^ were assigned to the D and G bands, respectively. Similar spectral features were also observed for the measurements performed using the 785 nm excitation laser (Figure S3, Supplementary Materials), confirming the presence of defect-containing graphitic domains in the g-C_3_N_4_/rGO nanocomposite. The increased relative intensity of the D band in the composite suggests a higher concentration of structural defects and disordered sp^2^-hybridized carbon domains originating from rGO. The calculated ID/IG ratios obtained from both excitation wavelengths are summarized in Figure S4 (Supplementary Materials). In all cases, the composite exhibited comparable or higher ID/IG values than pristine g-C_3_N_4_, supporting the introduction of defect-rich carbon structures associated with reduced graphene oxide. Furthermore, a weak and broad feature was observed in the 2D band region (~2700 cm^−1^), which is characteristic of disordered multilayer graphene-like structures rather than highly crystalline graphene sheets. These observations are consistent with the XRD, SEM, BET, and UV–Vis results, collectively confirming the successful incorporation of rGO into the g-C_3_N_4_ matrix. The presence of defect-rich carbon domains and the intimate contact between both components may facilitate charge carrier separation and interfacial electron transfer, contributing to the enhanced photocatalytic performance observed for the g-C_3_N_4_/rGO nanocomposite.

#### 2.2.5. Analysis of Scanning Electron Microscopy (SEM) Results

**Figure 4:**
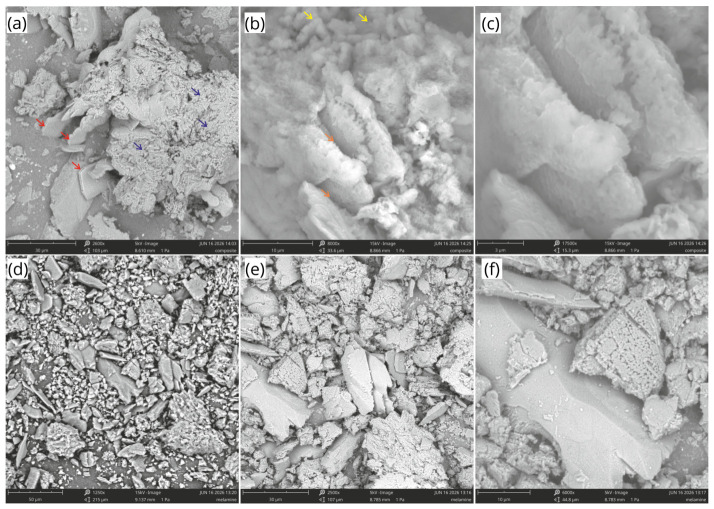
Representative SEM micrographs of the g-C_3_N_4_/rGO nanocomposite (**a**–**c**) and pristine g-C_3_N_4_ (**d**–**f**) showing the surface morphology and microstructural features of the investigated materials. Colored arrows highlight representative morphological features discussed in the text: plate-like particles (red), rough agglomerated regions (blue), wrinkled sheet-like structures (orange), and porous/corrugated surface regions (yellow).

The SEM micrographs of pristine g-C_3_N_4_ and the g-C_3_N_4_/rGO nanocomposite are presented in Figure 4. At lower magnification (Figure 4a), the composite exhibited a heterogeneous layered morphology composed of large wrinkled and overlapping sheet-like structures, forming an interconnected three-dimensional network. Numerous folds, cavities, and inter-sheet voids were observed throughout the material, indicating the formation of a highly developed surface architecture.

At intermediate magnification (Figure 4b), the composite revealed a combination of relatively smooth layered domains and more irregular porous regions. The folded sheet-like structures are consistent with the presence of graphene-derived layers, while the porous and flake-like features may be attributed to graphitic carbon nitride formed during thermal polycondensation. The intimate contact between these structural components suggests effective integration of both phases within the composite.

At higher magnification (Figure 4c), the material displayed a porous interconnected framework with thin layered structures distributed throughout the observed area. No obvious phase segregation was detected, indicating a relatively homogeneous morphology of the synthesized composite.

For comparison, SEM images of pristine g-C_3_N_4_ obtained from thermally treated melamine are shown in Figure 4d–f. In contrast to the composite, the reference material exhibited a more compact morphology consisting of agglomerated particles and larger block-like structures. Although porous regions were also observed, the material appeared more densely packed and less structurally developed than the g-C_3_N_4_/rGO composite.

The incorporation of rGO therefore resulted in a noticeable modification of the material morphology, leading to a more open, wrinkled, and interconnected structure. Such morphological changes are expected to improve the accessibility of active sites and facilitate mass transfer during photocatalytic reactions. These observations are consistent with the textural properties determined by BET analysis.

#### 2.2.6. Analysis of UV–Vis Spectroscopy Results

**Figure 5:**
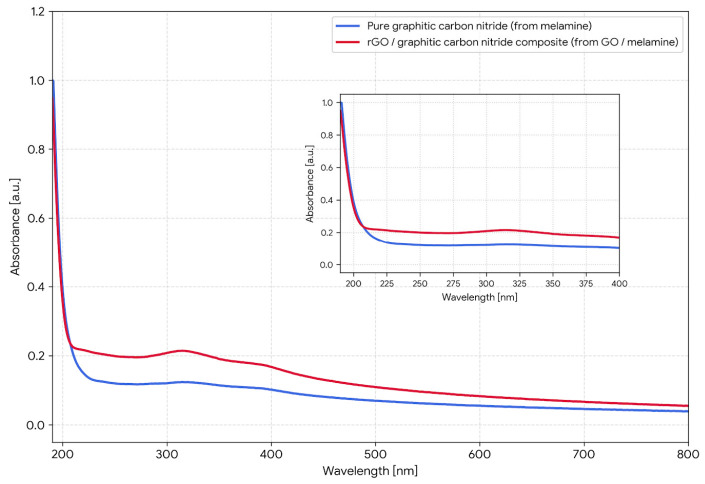
UV–Vis absorption spectra of pristine g-C_3_N_4_ and the g-C_3_N_4_/rGO nanocomposite recorded in the wavelength range of 190–800 nm. The inset shows an enlarged view of the UV region (190–400 nm).

The UV–Vis absorption spectra of pristine g-C_3_N_4_ and the synthesized g-C_3_N_4_/rGO composite are presented in Figure 5. The absorption edge and the broad feature observed at approximately 316 nm represent the most pronounced spectral difference between the two samples. The spectrum of pristine g-C_3_N_4_ exhibits a relatively flat and poorly defined plateau in this region, whereas the g-C_3_N_4_/rGO composite displays a broader and more distinct absorption maximum centered at approximately 316 nm. This absorption feature has been frequently associated with n → π* electronic transitions involving lone-pair electrons of nitrogen atoms within the triazine/heptazine units of graphitic carbon nitride [40,41].

At wavelengths above approximately 230 nm, the absorption spectrum of the g-C_3_N_4_/rGO composite remains consistently higher than that of pristine g-C_3_N_4_ throughout the investigated spectral range. The observed increase in absorbance may be associated with the presence of graphitic carbon domains originating from the reduced graphene oxide component and indicates enhanced overall light absorption by the composite material.

In the deep UV region (190–220 nm), both spectra show a rapid increase in absorbance, reaching values close to 1.0 at 190 nm and exhibiting very similar spectral profiles. These absorption features are typically assigned to π → π* electronic transitions within conjugated C=N and C=C bonds [42]. The similarity of both spectra in this region suggests that the incorporation of rGO did not substantially alter the fundamental electronic structure of the g-C_3_N_4_ framework.

The spectra intersect at approximately 208 nm, above which the absorbance of the g-C_3_N_4_/rGO composite remains consistently higher than that of pristine g-C_3_N_4_. This observation further confirms that the incorporation of rGO modifies the optical absorption characteristics of the material over a broad wavelength range.

Overall, the UV–Vis results indicate that the incorporation of rGO influences the optical properties of graphitic carbon nitride and contributes to increased light absorption over a broad spectral range. These observations are consistent with the improved photocatalytic performance observed during the degradation experiments.

### 2.3. Photocatalytic Degradation Using g-C_3_N_4_/rGO Nanocomposite

#### 2.3.1. Degradation Kinetics of Pharmaceuticals

**Figure 6:**
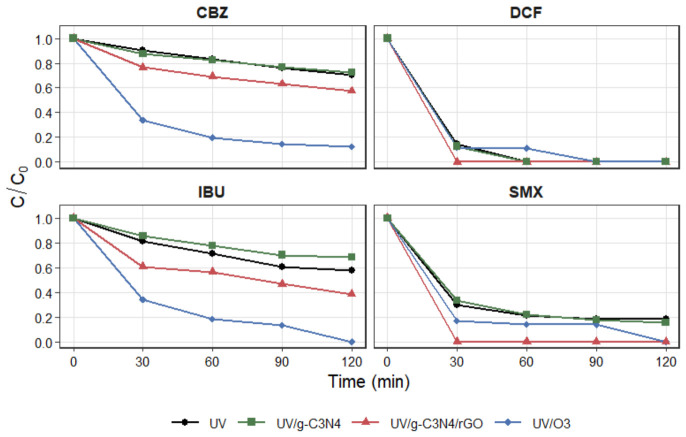
Normalized concentration profiles (C/C_0_) of sulfamethoxazole (SMX), carbamazepine (CBZ), diclofenac (DCF), and ibuprofen (IBU) during UV photolysis, UV/O_3_, UV/g-C_3_N_4_, and UV/g-C_3_N_4_/rGO photocatalytic treatment conducted in MBR-treated wastewater.

The photocatalytic activity of the synthesized g-C_3_N_4_/rGO nanocomposite was evaluated toward the degradation of selected pharmaceuticals in MBR-treated wastewater under UV irradiation. Changes in the normalized concentration ratio (C/C_0_) during the process are presented in Figure 6, while the calculated pseudo-first-order kinetic parameters are summarized in Table 3. Detailed regression statistics and raw degradation data are provided in the Supplementary Materials (Tables S1 and S2, respectively). The obtained results confirmed that the photocatalytic system exhibited compound-dependent degradation performance, strongly influenced by the physicochemical properties and photoreactivity of the investigated pharmaceuticals.

Among the analyzed compounds, DCF and sulfamethoxazole SMX showed the highest susceptibility toward photocatalytic degradation. Both compounds were rapidly removed from the wastewater matrix, reaching concentrations below the detection limit within the initial stage of the process. Consequently, the determination of reliable kinetic constants for these pharmaceuticals was not possible due to the insufficient number of measurable experimental points. The rapid degradation of DCF may be associated with its high photosensitivity and strong susceptibility to oxidation by reactive oxygen species (ROS), particularly hydroxyl radicals (•OH) and superoxide radicals (•O_2_^−^), generated on the surface of the irradiated photocatalyst [43]. Similar behavior has been frequently reported for aromatic anti-inflammatory drugs containing electron-donating functional groups that promote electrophilic attack and ring-opening reactions during photocatalytic oxidation [44].

A similarly high degradation efficiency was observed for SMX, which also exhibited complete removal within the first sampling interval. Sulfonamide antibiotics are known to undergo efficient photocatalytic transformation due to the presence of electron-rich amino groups and heterocyclic structures susceptible to oxidative degradation [45]. The obtained results indicate that the synthesized g-C_3_N_4_/rGO nanocomposite may have facilitated reactive oxygen species generation despite the presence of residual organic and inorganic constituents in the wastewater matrix, which could compete for reactive species and consequently reduce degradation efficiency [32].

In contrast, CBZ exhibited significantly higher resistance toward photocatalytic oxidation. After 120 min of irradiation, the RE reached approximately 42.6%, indicating only moderate degradation under the applied conditions. The calculated pseudo-first-order rate constant for CBZ was 0.0044 min^−1^, while the corresponding half-life time exceeded 150 min. The relatively low degradation efficiency of CBZ is consistent with previous reports describing this compound as one of the most recalcitrant pharmaceutical micropollutants commonly detected in treated wastewater effluents [46,47]. Its high stability is primarily related to the fused aromatic structure and low reactivity toward hydroxyl radicals, which limit the overall oxidation rate.

IBU demonstrated intermediate degradation behavior compared with the other investigated pharmaceuticals. The removal efficiency reached approximately 61.5% after 120 min of treatment, corresponding to a pseudo-first-order rate constant of 0.0073 min^−1^. Although the degradation efficiency was noticeably lower than that observed for DCF and SMX, the photocatalytic system still enhanced IBU removal compared with UV photolysis alone. This observation suggests that the presence of rGO may contribute to facilitated electron transport and reduced charge carrier recombination within the photocatalytic system [32].

Overall, the obtained results confirmed that the synthesized g-C_3_N_4_/rGO nanocomposite was capable of enhancing the degradation of selected pharmaceuticals in a realistic wastewater matrix. However, the degradation efficiency strongly depended on the molecular structure and oxidation susceptibility of the investigated compounds. The observed differences between pharmaceuticals additionally indicate that matrix effects and ROS scavenging phenomena likely played an important role during the photocatalytic process conducted in MBR-treated wastewater.

#### 2.3.2. Mechanistic Aspects of Photocatalytic Degradation

The photocatalytic degradation process occurring in the UV/g-C_3_N_4_/rGO system was primarily associated with the generation of reactive oxygen species (ROS) formed during irradiation of the nanocomposite surface. Upon UV excitation, electrons from the valence band of g-C_3_N_4_ were promoted to the conduction band, resulting in the formation of electron–hole pairs (e^−^/h^+^). These charge carriers subsequently participated in oxidation–reduction reactions leading to the formation of highly reactive species, including hydroxyl radicals (•OH) and superoxide radicals (•O_2_^−^), which are considered the major oxidizing agents responsible for pharmaceutical degradation. Similar ROS-mediated degradation pathways have been widely described for g-C_3_N_4_-based photocatalytic systems applied for wastewater treatment [27,48].

Hydroxyl radicals are characterized by extremely high oxidation potential and non-selective reactivity toward organic contaminants. Consequently, DCF and SMX, containing electron-rich aromatic structures and functional groups susceptible to electrophilic attack, underwent rapid degradation during the photocatalytic process. In contrast, CBZ exhibited substantially lower degradation efficiency, indicating reduced susceptibility toward ROS-mediated oxidation under the applied experimental conditions. The relatively high persistence of CBZ observed in the present study is consistent with previous reports identifying this compound as one of the most recalcitrant pharmaceutical micropollutants frequently detected in treated wastewater effluents [49].

The presence of rGO within the composite structure may contribute to facilitated electron transport and improved separation of photogenerated charge carriers, as suggested in previous studies on carbon-modified g-C_3_N_4_ systems [28]. Due to its high electrical conductivity and electron-accepting properties, rGO acted as an electron transport mediator, facilitating charge carrier separation and suppressing rapid electron–hole recombination. As a result, a greater number of photogenerated electrons remained available for reactions with dissolved oxygen, promoting the formation of superoxide radicals (•O_2_^−^) and enhancing overall ROS generation. Similar improvements in photocatalytic efficiency after incorporation of conductive carbon-based materials into g-C_3_N_4_ systems have been widely reported in the literature [28]. Similar observations regarding the role of carbon-based composite photocatalysts in enhancing photocatalytic degradation under complex aqueous conditions have also been reported in recent studies [30,31].

The improved charge separation properties associated with the presence of rGO likely contributed to the enhanced degradation efficiency observed for ibuprofen (IBU) and carbamazepine compared with UV photolysis alone. Although complete mineralization was not achieved within the investigated reaction time, the photocatalytic system noticeably accelerated the degradation kinetics of the more persistent compounds, indicating that the synthesized nanocomposite effectively enhanced ROS availability under irradiation conditions.

Nevertheless, the photocatalytic experiments were performed in real MBR-treated wastewater rather than in ultrapure water, introducing additional complexity to the degradation mechanism. Residual dissolved organic matter, inorganic ions, and carbonate species present in the treated effluent may compete with target pharmaceuticals for reactive oxygen species and active sites on the photocatalyst surface. The MBR-treated wastewater used in this study was characterized by residual organic matter (COD 38.20–39.13 mg O_2_/L; BOD_5_ 5–6 mg O_2_/L), chloride ions (143–145 mg/L), and inorganic nitrogen species, including nitrate (3.06–3.31 mg/L), nitrite (0.076–0.088 mg/L), and ammonium (0.24–0.27 mg/L). These constituents may interact with reactive oxygen species generated during photocatalysis and reduce their availability for pharmaceutical degradation. In particular, chloride ions can react with hydroxyl radicals (•OH), forming less reactive chlorine-based radical species, while residual organic matter may act as a competitive scavenger of oxidizing radicals. Consequently, the complex wastewater matrix may partially suppress photocatalytic efficiency compared with simplified model systems. Additionally, carbonate and bicarbonate species, commonly present in municipal wastewater, are known scavengers of hydroxyl radicals, leading to the formation of carbonate radicals (CO_3_•^−^), which exhibit lower oxidation potential and higher selectivity toward organic compounds. Such scavenging effects are considered one of the major factors influencing photocatalytic treatment efficiency in real wastewater matrices compared with experiments conducted in ultrapure water [18,48]. However, carbonate and bicarbonate concentrations were not quantified in the present study and their individual contribution to the observed degradation behavior could not be determined.

Despite these matrix-related limitations, the synthesized g-C_3_N_4_/rGO nanocomposite maintained photocatalytic activity in the real wastewater matrix, confirming its potential applicability as a post-treatment technology for MBR effluents. Additional control experiments performed with pristine g-C_3_N_4_ under identical operating conditions revealed that the incorporation of rGO was responsible for the observed enhancement in photocatalytic performance. While pristine g-C_3_N_4_ exhibited removal efficiencies comparable to or lower than those achieved by UV photolysis alone for selected pharmaceuticals, the g-C_3_N_4_/rGO nanocomposite provided improved degradation, particularly in the case of carbamazepine and ibuprofen. The obtained results additionally demonstrate the importance of evaluating photocatalytic materials under environmentally relevant conditions, since degradation efficiencies obtained in ultrapure water may substantially differ from those observed in real wastewater systems due to radical scavenging and competitive interactions. However, the present study did not include quantitative analysis of individual radical scavengers or comparative experiments in ultrapure water; therefore, the exact contribution of specific wastewater matrix constituents to the observed inhibition effects could not be fully determined.

### 2.4. Comparative Evaluation of Treatment Processes

**Figure 7:**
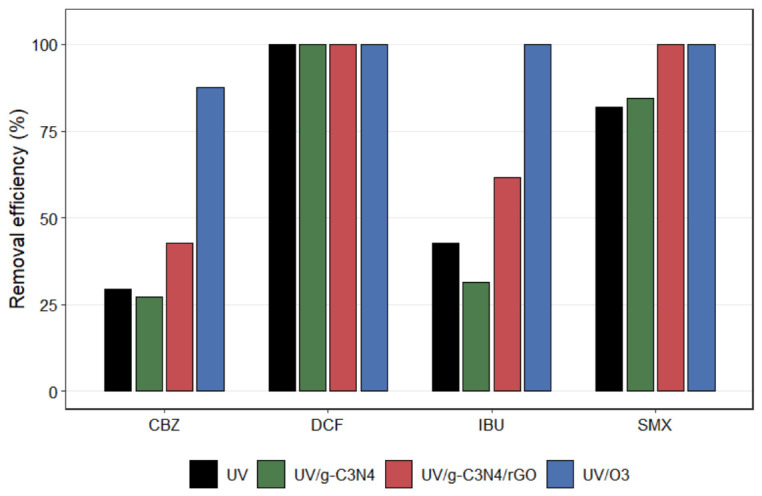
Comparison of the final removal efficiencies of sulfamethoxazole (SMX), carbamazepine (CBZ), diclofenac (DCF), and ibuprofen (IBU) after 120 min of treatment using UV photolysis, UV/O_3_, UV/g-C_3_N_4_ and UV/g-C_3_N_4_/rGO photocatalytic processes in MBR-treated wastewater.

The comparative analysis of UV photolysis, UV/O_3_ oxidation, and photocatalytic treatment using the synthesized g-C_3_N_4_/rGO nanocomposite demonstrated substantial differences in degradation efficiency depending on the applied process and pharmaceutical compound (Figure 6 and Figure 7, Table 3). Among the investigated systems, the UV/O_3_ process exhibited the highest overall degradation performance, resulting in complete removal of DCF, SMX, and IBU, as well as the highest elimination rate for carbamazepine (CBZ). The enhanced performance of the UV/O_3_ system was associated with the intensive generation of hydroxyl radicals (•OH) originating from simultaneous ozone decomposition and UV irradiation. This synergistic effect, primarily driven by the photolysis of ozone to hydrogen peroxide and its subsequent cleavage into •OH, leads to rapid oxidation of pharmaceutical compounds even in the presence of wastewater matrix constituents [50].

In contrast, direct UV photolysis showed considerably lower removal kinetics, particularly for CBZ and IBU, confirming the limited susceptibility of these compounds to direct photochemical transformation. This resistance is generally attributed to their low molar absorption coefficients at the applied UV wavelength (e.g., 254 nm) and relatively low quantum yields of photodegradation [51]. Although complete degradation of DCF was observed during UV treatment, the process remained less effective overall than UV/O_3_ and exhibited significantly lower kinetic constants for the more recalcitrant pharmaceuticals.

The UV/g-C_3_N_4_/rGO photocatalytic system demonstrated intermediate degradation performance between direct UV photolysis and UV/O_3_ oxidation. Although the observed improvement compared with UV irradiation alone was moderate, particularly for ibuprofen and carbamazepine, the obtained results indicate the potential applicability of the investigated nanocomposite under realistic wastewater conditions. The observed differences in degradation efficiency may be partially associated with the presence of conductive carbon-based components facilitating interfacial electron transfer processes under irradiation conditions.

Importantly, although the UV/O_3_ process provided superior degradation efficiency, its practical large-scale application may be associated with several operational and environmental limitations. These include high energy demand, continuous ozone generation requirements, elevated operational costs, and the potential formation of toxic oxidation by-products, such as bromate (BrO_3_^−^) in bromide-containing matrices or toxic intermediates resulting from dissolved organic matter (DOM) oxidation [52]. Consequently, increasing attention has been directed toward the development of more sustainable and environmentally friendly treatment technologies capable of reducing energy consumption and chemical input while maintaining satisfactory removal performance.

In this context, the investigated g-C_3_N_4_/rGO nanocomposite appears to be a promising alternative or complementary post-treatment strategy for MBR effluents. The photocatalytic system operated without external oxidant addition and was based on a metal-free photocatalyst characterized by relatively low toxicity and high chemical stability. Utilizing metal-free carbon-based semiconductors eliminates the risk of secondary pollution caused by transition metal leaching, a major drawback frequently disqualifying conventional catalysts in practical wastewater treatment [53]. Moreover, the ability of the nanocomposite to maintain photocatalytic activity in a realistic wastewater matrix suggests its potential applicability under practical treatment conditions. Although the degradation efficiency remained lower than that achieved by UV/O_3_, the photocatalytic approach may offer advantages related to lower environmental impact, reduced chemical consumption, and improved process sustainability.

Overall, the obtained results indicate that the integration of advanced biological treatment systems such as MBRs with photocatalytic post-treatment based on g-C_3_N_4_/rGO nanocomposites may represent a promising direction for the removal of persistent pharmaceutical micropollutants from wastewater. Nevertheless, further studies should focus on process optimization, catalyst stability, long-term performance evaluation, transformation product identification, and toxicity assessment in order to fully evaluate the practical applicability of the proposed treatment strategy.

## 3. Materials and Methods

### 3.1. Chemicals and Reagents

All pharmaceutical compounds (PhCs) used in the analysis were analytical-grade substances of high purity: diclofenac sodium salt (purity ≥ 98%, TLC, Sigma-Aldrich, St. Louis, MO, USA), ibuprofen sodium salt (purity ≥ 98%, GC, Sigma-Aldrich, St. Louis, MO, USA), sulfamethoxazole (GC, HPLC, Sigma-Aldrich, St. Louis, MO, USA) and carbamazepine (purity ≥ 97.0%, Glentham Life Sciences, Corsham, UK). Reagents such as ethanol (≥99.9%), ultra-pure water (for HPLC), acetonitrile (≥99.9%), phosphoric acid (H_3_PO_4_), and melamine were purchased at Sigma-Aldrich (St. Louis, MO, USA). Reduced graphene oxide (rGO) was purchased at Institute of Carbon Technologies (Toruń, Poland). The stock solution was prepared by dissolving 500 mg of each of the four PhCs in 10 mL of methanol, resulting in a 50 mg/mL concentration for each PhCs. The stock solution was stored at 2–6 degrees Celsius. The research used a working solution with a concentration of 50 mg/L of each pharmaceutical obtained by diluting the stock solution a thousand times with effluent wastewater. The applied pharmaceutical concentration (50 mg/L) was selected to ensure reliable analytical quantification and kinetic evaluation under controlled laboratory conditions, as commonly reported in photocatalytic studies [54,55].

### 3.2. Wastewater Sampling and Characterization

Wastewater samples were collected from a municipal wastewater treatment plant (15,000 population equivalent (PE)) equipped with a Membrane Biological Reactor (MBR) system (Schwander Polska, Podegrodzie, Poland). Treated effluent samples were obtained at the outlet of the treatment facility using an automatic sampler. Composite samples were collected at 1-h intervals over a 24-h period to ensure representative characterization of the effluent. Immediately after collection, samples were subjected to further analysis. In cases where storage was necessary, samples were kept at 4 °C in light-impermeable containers to prevent degradation of target compounds and minimize potential changes in physicochemical properties.

#### Physicochemical Parameters

Basic physicochemical parameters of the collected MBR-treated wastewater samples were determined to characterize the effluent matrix used in the photocatalytic experiments. The analyzed parameters included pH, electrical conductivity, total organic carbon (TOC), and chemical oxygen demand (COD), which are commonly used to assess the composition and organic load of wastewater effluents. The obtained values were intended to evaluate the potential influence of the wastewater matrix on photocatalytic degradation processes, particularly with respect to radical scavenging phenomena and competitive interactions between wastewater constituents and target pharmaceuticals. All measurements were performed using standard analytical methods. The physicochemical characteristics of the treated wastewater are summarized in Table 1.

### 3.3. Synthesis and Characterization of g-C_3_N_4_/rGO Nanocomposite

#### 3.3.1. Synthesis of g-C_3_N_4_/rGO Nanocomposite

The g-C_3_N_4_/rGO nanocomposite was synthesized via a one-step thermal polycondensation method, enabling simultaneous formation of graphitic carbon nitride and in situ reduction of graphene oxide.

In this procedure, 10.00 g of melamine (≥99%, Sigma-Aldrich, St. Louis, MO, USA) and 0.10 g of reduced graphene oxide (rGO, Institute of Carbon Technologies, Toruń, Polska) were accurately weighed. The precursors were thoroughly mixed to obtain a homogeneous mixture. The prepared mixture was transferred into a ceramic crucible with a fitted lid, which was essential to limit the loss of volatile intermediates and ensure controlled condensation. The crucible was placed in a muffle furnace and heated in air at a rate of 2–3 °C/min up to 550 °C, followed by a holding time of 3 h. After the thermal treatment, the furnace was switched off, and the sample was allowed to cool naturally inside the furnace to approximately 100 °C.

The obtained yellowish powder was gently ground and sieved (100 μm mesh screen) to ensure uniform particle size.

The synthesis parameters, including temperature (550–610 °C), heating rate (5–10 °C/min), and calcination time (2–4 h), were selected based on literature reports to ensure optimal structural and photocatalytic properties of the obtained nanocomposite [56]. The synthesized g-C_3_N_4_/rGO nanocomposite was subsequently characterized using FTIR–ATR spectroscopy, X-ray diffraction (XRD), and UV–Vis spectroscopy to evaluate its structural and optical properties.

#### 3.3.2. FTIR–ATR Analysis

Fourier-transform infrared (FTIR) spectroscopy analysis was performed using a FTIR spectrophotometer (Shimadzu IRaffinity-1S, Kyoto, Japan) equipped with an attenuated total reflectance (ATR) accessory with a diamond crystal. Spectra were recorded over the wavenumber range of 400–4000 cm^−1^. Each spectrum was obtained by co-averaging 128 scans at a spectral resolution of 2 cm^−1^. All samples were re-measured to verify reproducibility and ensure spectral accuracy.

#### 3.3.3. X-Ray Diffraction Analysis

X-ray diffraction (XRD) measurements were performed on powdered samples of pristine g-C_3_N_4_ and g-C_3_N_4_/rGO nanocomposite using a Bruker D8 Advance diffractometer (Bruker AXS, Karlsruhe, Germany) equipped with Cu Kα radiation (λ = 1.5406 Å). Diffraction patterns were recorded over a 2θ range of 5–80° with a step size of 0.0242°. Phase identification was carried out using interplanar spacing values calculated according to Bragg’s law and by comparison with reference patterns from the ICDD PDF-2 database. The diffraction patterns were interpreted using literature data and reference patterns corresponding to graphitic carbon nitride (g-C_3_N_4_), graphitic carbon phases associated with reduced graphene oxide (rGO), and melamine-derived intermediate species. Additional details regarding diffraction peak deconvolution and phase identification are provided in the Supplementary Materials.

#### 3.3.4. Surface Area and Porosity Analysis (BET)

The textural properties of pristine g-C_3_N_4_ and the g-C_3_N_4_/rGO nanocomposite were determined by nitrogen adsorption–desorption measurements at 77 K using a Micromeritics 3Flex surface characterization analyzer. The specific surface area was calculated according to the Brunauer–Emmett–Teller (BET) method. Mesopore volume and pore size distribution were determined from the adsorption branch of the isotherm using the Barrett–Joyner–Halenda (BJH) model. Micropore characteristics were evaluated using t-plot analysis and the Horváth–Kawazoe (HK) method. Prior to analysis, the samples were degassed under vacuum according to the instrument operating procedure. Detailed adsorption–desorption isotherms and pore size distribution data are provided in the Supplementary Materials.

#### 3.3.5. Raman Spectroscopy

Raman spectra were collected using a WITec alpha300 RA Raman microscope (WITec GmbH, Ulm, Germany) equipped with a confocal optical system. Powdered samples were deposited onto a stainless-steel sample holder, previously verified to be Raman inactive within the investigated spectral range. The powders were gently pressed with a glass slide to obtain a flat and homogeneous measurement surface.

Measurements were performed using two excitation wavelengths, 532 nm and 785 nm, in order to evaluate the influence of laser wavelength on spectral quality and fluorescence suppression. For each sample, the laser power and acquisition time were individually optimized to maximize the signal-to-noise ratio while avoiding signal distortion and excessive fluorescence. All measurements were carried out using a 50× objective lens. Raman spectra were collected from at least two different locations on each sample to assess spectral reproducibility and sample homogeneity. Raw spectra were subsequently subjected to baseline correction and normalization prior to comparative analysis.

#### 3.3.6. Scanning Electron Microscopy (SEM)

SEM was employed to investigate the morphology and surface characteristics of pristine g-C_3_N_4_ and the synthesized g-C_3_N_4_/rGO nanocomposite, as well as to evaluate the effect of rGO incorporation on the structural organization of the material. SEM images were acquired using a Phenom ProX scanning electron microscope (Phenom-World, The Netherlands) operated at accelerating voltages of 5 and 15 kV under low-vacuum conditions (1 Pa). Micrographs were collected at various magnifications to assess particle morphology, surface texture, agglomeration behavior, and morphological changes associated with the incorporation of rGO into the g-C_3_N_4_ framework.

#### 3.3.7. UV–Vis Spectroscopy

Aqueous dispersions of the synthesized g-C_3_N_4_/rGO nanocomposite and pristine g-C_3_N_4_ were prepared at a concentration of 0.15 mg mL^−1^ by dispersing 1.5 mg of material in 10 mL of demineralized water. To improve dispersion stability, a small amount of NH_4_OH solution was added to obtain slightly alkaline conditions. The same procedure was applied to the pristine g-C_3_N_4_ sample used as a reference.

The suspensions were sonicated for 60 min in an ultrasonic bath under external cooling and subsequently centrifuged at 5000 rpm for 10 min to remove larger agglomerates. The resulting supernatants were used for spectroscopic measurements.

Optical absorption measurements were performed using a Cary 100 Bio UV–Vis spectrophotometer (Agilent Technologies, Santa Clara, CA, USA). Spectra were recorded in the wavelength range of 190–800 nm with a step size of 1 nm using quartz cuvettes. Instrumental background correction was performed prior to analysis. The obtained spectra were used for comparative evaluation of the optical absorption properties of pristine g-C_3_N_4_ and the g-C_3_N_4_/rGO composite.

### 3.4. Experimental Procedures

#### 3.4.1. Photolysis and UV/Ozonation Process

We carried out photolysis and a UV/ozonation process using a laboratory-scale UV reactor system (UV Reactor System 3, Heraeus, Hanau, Germany) equipped with a low-pressure mercury immersion lamp (TNN 15/32, principal emission at ~254 nm), housed in a quartz sleeve and installed in a jacketed reactor vessel. All experiments were performed in batch mode with a working volume of 700 mL, under simultaneous exposure to UV radiation and ozone. The process conditions were maintained constant throughout the experiments to ensure reproducibility. Ozone was produced from atmospheric air using a laboratory-scale ozone generator. The gas flow rate was maintained at 5.0 L·min^−1^ and controlled using a rotameter. Ozone was continuously introduced into the reaction mixture through a porous diffuser positioned at the bottom of the reactor to ensure effective gas dispersion and contact with the treated solution.

Samples were collected at predetermined time intervals (30, 60, 90, and 120 min). Each sample was withdrawn using a syringe and immediately filtered through a membrane 0.22 μg PES filter prior to HPLC analysis.

#### 3.4.2. Photocatalytic Process Using g-C_3_N_4_/rGO

Photocatalytic experiments were conducted in batch mode using the laboratory-scale UV reactor system (UV Reactor System 3, Heraeus, Hanau, Germany) equipped with a low-pressure mercury immersion lamp (TNN 15/32, principal emission at ~254 nm), identical to that used in the photolysis and UV/O_3_ experiments. The synthesized g-C_3_N_4_/rGO nanocomposite was applied in suspended form at a concentration of 0.3 g·L^−1^ during the photocatalytic process. The working volume of the treated solution was 700 mL, and the initial concentration of each target pharmaceutical compound was 50 mg·L^−1^.

Prior to UV irradiation, the suspension containing the photocatalyst was maintained in the dark for 30 min to establish adsorption–desorption equilibrium between the catalyst surface and the pharmaceutical compounds. Subsequently, the photocatalytic process was initiated by switching on the UV lamp.

The reaction time and sampling intervals were consistent with those applied in the UV and UV/O_3_ experiments to enable direct comparison between the investigated processes. For UV photolysis experiments, ozone generation was switched off and all remaining operating conditions were kept identical. Samples were collected at predetermined time intervals (30, 60, 90, and 120 min) using a syringe and immediately filtered through a 0.22 µm PES membrane filter prior to chromatographic analysis.

### 3.5. Analytical Methods and Chromatographic Conditions

#### 3.5.1. Instrumentation

The concentrations of selected pharmaceuticals were determined using high-performance liquid chromatography (HP, LC) equipped with a diode array detector (DAD) (Thermo Scientific, Waltham, MA, USA). Separation was carried out on a reversed-phase analytical column (Accucore™ C18, 2.6 µm, 150 × 3 mm). Data acquisition and processing were performed using Chromeleon™ 7 CDS software (Thermo Fisher Scientific, Waltham, MA, USA).

#### 3.5.2. Chromatographic Conditions

Chromatographic separation was performed at a flow rate of 0.6 mL·min^−1^, with the column temperature maintained at 40 °C. Detection was carried out at a wavelength of 230 nm.

The mobile phase consisted of two components: (A) ultrapure water acidified with phosphoric acid to pH 2.8 and (B) acetonitrile. A gradient elution program was applied to ensure effective separation of the target compounds. Initially, the system was operated with a high proportion of aqueous phase (90% A) for 2 min. Subsequently, the proportion of organic solvent (B) was increased and maintained at a high level for the next 17 min. In the final stage of the analysis, the mobile phase composition was returned to initial conditions. The total analysis time per sample was 21 min. The applied chromatographic method was developed based on literature data and optimized for the simultaneous determination of the selected pharmaceuticals.

### 3.6. Data Analysis

The photocatalytic degradation of the target pharmaceuticals was monitored by tracking the normalized concentration ratio C/C_0_, where C_0_ denotes the initial concentration of the pharmaceutical compound and C represents the concentration measured at time t during irradiation. The removal efficiency (RE) was calculated according to Equation (1):

(1) $$RE\;[\%]=\frac{C_{0}-C}{C_{0}}\times 100$$

where C_0_ is the initial concentration of the pharmaceutical compound (mg·L^−1^) and C is the concentration remaining at time t (mg·L^−1^).

To evaluate the reaction kinetics, a pseudo-first-order kinetic model was applied, as described by Equation (2):

(2) $$ln\left(\frac{C_{0}}{C}\right)=kt$$

where k is the pseudo-first-order rate constant (min^−1^) and t is the irradiation time (min). The kinetic constants were determined from the slopes of linear regressions obtained for ln(C_0_/C) as a function of irradiation time. Coefficients of determination (R^2^) and additional regression statistics were calculated to evaluate the fitting quality of the applied kinetic model.

Due to the exploratory character of the study and the preliminary assessment of the photocatalytic performance of the synthesized nanocomposite under realistic wastewater conditions, the experiments were conducted as single-batch runs. Therefore, the data interpretation was primarily based on comparative degradation trends, removal efficiencies, and kinetic analysis rather than inferential statistical testing.

All calculations and graphical representations were performed using Microsoft Excel (Microsoft 365, Microsoft Corporation, Redmond, WA, USA) and R software (version 4.2.2, R Foundation for Statistical Computing, Vienna, Austria).

## 4. Conclusions

The present study demonstrated that the synthesized g-C_3_N_4_/rGO nanocomposite was capable of supporting the photocatalytic degradation of selected pharmaceutical compounds in real MBR-treated wastewater under UV irradiation. Complete removal of diclofenac and sulfamethoxazole was achieved within 30 min, while the nanocomposite enhanced the degradation efficiency of ibuprofen and carbamazepine by approximately 19% and 13%, respectively, compared to UV irradiation alone. The obtained results indicate the potential applicability of the investigated photocatalytic system as a preliminary post-treatment approach for pharmaceutical removal from biologically treated wastewater.

Comprehensive physicochemical characterization confirmed the successful incorporation of rGO into the g-C_3_N_4_ matrix. XRD analysis revealed the characteristic structural features of graphitic carbon nitride and the presence of graphitic carbon domains associated with rGO, while Raman spectroscopy confirmed the formation of defect-rich sp^2^ carbon structures. UV–Vis measurements demonstrated enhanced light absorption of the composite over a broad wavelength range. Furthermore, BET analysis showed a slight increase in specific surface area and pore volume after rGO incorporation, together with the appearance of microporosity, whereas SEM observations revealed a layered and interconnected morphology that may facilitate mass transfer and photocatalytic reactions.

The experiments conducted in real wastewater rather than model solutions increase the practical relevance of the findings, although matrix components such as dissolved organic matter, chloride ions, and carbonate species may influence degradation kinetics through radical scavenging and competitive interactions. Despite these limitations, the g-C_3_N_4_/rGO nanocomposite maintained photocatalytic activity under environmentally relevant conditions, suggesting that coupling MBR systems with g-C_3_N_4_/rGO-based photocatalysis may represent a promising post-treatment strategy for the removal of persistent pharmaceutical micropollutants.

Several limitations should be acknowledged. The present study focused primarily on the removal of selected parent pharmaceutical compounds and did not include the identification of transformation products, total organic carbon (TOC) measurements, or ecotoxicological assessment of treated effluents. Consequently, the extent of mineralization and the potential environmental impact of degradation intermediates could not be evaluated. In addition, catalyst stability, reusability, and post-reaction structural characterization were beyond the scope of the current work.

Future studies should therefore focus on catalyst stability and recovery, reusability assessment, identification of degradation intermediates, toxicity evaluation of treated wastewater, and optimization of operational parameters to support the development of scalable and environmentally sustainable photocatalytic treatment systems.

## Figures and Tables

**Table 2:** Textural properties of pristine g-C_3_N_4_ and the g-C_3_N_4_/rGO nanocomposite determined from N_2_ adsorption–desorption measurements.

Sample	BET Surface Area (m^2^/g)	Micropore Volume (cm^3^/g)	Median Micropore Width (nm)	BJH Pore Volume (cm^3^/g)
**g-C_3_N_4_**	10.17	n.d.	n.d.	0.0637
**g-C_3_N_4_/rGO**	11.49	0.0033	1.16	0.0794

n.d.—not detected (no measurable microporosity was identified by t-plot analysis).

**Table 3:** Summary of pseudo-first-order kinetic parameters for the degradation of pharmaceuticals during UV, UV/O_3_, and UV/g-C_3_N_4_/rGO treatment processes in MBR-treated wastewater.

Process	Compound	k_obs_ [min^−1^]	R^2^	t_1/2_ [min]
UV/O_3_	SMX	0.0206	0.6878	33.72
UV/O_3_	CBZ	0.0167	0.8824	41.58
UV/O_3_	DCF	0.0364	0.7579	19.07
UV/O_3_	IBU	0.0221	0.9348	31.32
UV	SMX	0.0130	0.7370	53.51
UV	CBZ	0.0029	0.9968	238.88
UV	DCF	n.d.	-	-
UV	IBU	0.0047	0.9666	147.85
UV/g-C_3_N_4_	SMX	0.0146	0.8392	47.50
UV/g-C_3_N_4_	CBZ	0.0026	0.9645	271.84
UV/g-C_3_N_4_	DCF	n.d.	-	-
UV/g-C_3_N_4_	IBU	0.0032	0.9440	217.89
UV/g-C_3_N_4_/rGO	SMX	n.d.	-	-
UV/g-C_3_N_4_/rGO	CBZ	0.0044	0.9335	159.38
UV/g-C_3_N_4_/rGO	DCF	n.d.	-	-
UV/g-C_3_N_4_/rGO	IBU	0.0073	0.9187	95.66

n.d.—not determined due to rapid degradation within the initial sampling interval.

## Data Availability

The data presented in this study are openly available in Zenodo at https://zenodo.org/ (accessed on 18 May 2026).

## Supplementary Materials

The following supporting information can be downloaded at: https://www.mdpi.com/article/10.3390/molecules31132346/s1, Figure S1: N_2_ adsorption–desorption isotherms of pristine g-C_3_N_4_; Figure S2: N_2_ adsorption–desorption isotherms of the g-C_3_N_4_/rGO nanocomposite; Figure S3: Mean baseline-corrected and normalized Raman spectra of pristine g-C_3_N_4_ and the g-C_3_N_4_/rGO nanocomposite recorded using a 785 nm excitation laser; Figure S4: D-to-G band intensity ratios (ID/IG) determined from baseline-corrected Raman spectra recorded at 532 and 785 nm excitation wavelengths for pristine g-C_3_N_4_ and the g-C_3_N_4_/rGO nanocomposite; Table S1: Raw concentration data obtained during the degradation of pharmaceuticals under UV, UV/O_3_, UV/g-C_3_N_4_, and UV/g-C_3_N_4_/rGO treatment processes; Table S2: Pseudo-first-order kinetic parameters calculated for the degradation of pharmaceuticals during UV, UV/O_3_, UV/g-C_3_N_4_, and UV/g-C_3_N_4_/rGO processes.
