# Supplementary material for: Enhanced Photocatalytic Removal of Selected Pharmaceuticals from MBR-Treated Wastewater Using a g-C3N4/rGO Nanocomposite Under UV Irradiation
Source: Molecules. 2026 Jul 3;31(13):2346. doi: 10.3390/molecules31132346 (PMC13363225; doi:10.3390/molecules31132346)
Supplement: Supplementary file 1 [file molecules-31-02346-s001.zip › molecules-4355878-supplementary.pdf]

Article

Enhanced Photocatalytic Removal of Selected Pharmaceuticals from MBR-Treated Wastewater Using a g-C_3N_4/rGO Nanocomposite Under UV Irradiation

Klaudia Całus-Makowska [1,*], Renata Caban [2], Robert Zarzycki [1], Tomasz Kamizela [1], Marcin Dośpiał [2] and Anna Grobelak [1]

[1] Faculty of Infrastructure and Environment, Czestochowa University of Technology, 42-200 Częstochowa, Poland; robert.zarzycki@pcz.pl (R.Z.); tomasz.kamizela@pcz.pl (T.K.); anna.grobelak@pcz.pl (A.G.)

[2] Faculty of Production Engineering and Materials Technology, Czestochowa University of Technology, 42-201 Czestochowa, Poland; renata.caban@pcz.pl (R.C.); marcin.dospial@pcz.pl (M.D.)

* Correspondence: k.calus-makowska@pcz.pl

Section S1. Characterization of g-C_3N_4/(r)GO nanocomposite

1. X-ray Diffraction (XRD) Analysis

X-ray diffraction (XRD) measurements were performed on powdered samples of the synthesized g-C_3N_4/rGO nanocomposite using a Bruker D8 Advance diffractometer (Bruker AXS, Germany) equipped with Cu Kα radiation (λ = 1.5406 Å). Diffraction patterns were collected over a 2θ range of 5–80° using a step size of 0.0242°.
Phase identification was conducted based on interplanar spacing values (d) calculated according to Bragg's law:

$$\lambda = 2d \sin \theta \quad (1)$$

where λ is the X-ray wavelength, d is the interplanar spacing, and θ is the Bragg angle.

The obtained diffraction profiles were compared with reference patterns from the ICDD PDF-2 database. Structural features associated with graphitic carbon nitride (g-C_3N_4) were assigned using reference card No. 00-066-0813, graphitic carbon structures related to reduced graphene oxide (rGO) were evaluated using card No. 00-041-1487 (Graphite-2H), while residual melamine was assessed using card No. 00-039-1950.

For a more detailed evaluation of broad diffraction features, diffraction profiles were subjected to Gaussian peak deconvolution. The experimental diffraction pattern was described as a sum of individual Gaussian functions:

$$I(2\theta) = \Sigma Ii(2\theta) \quad (2)$$

where Ii(2θ) represents the intensity contribution of each diffraction component.

The integrated area under each fitted peak was calculated and used for comparative evaluation of the relative contribution of individual structural domains. Peak fitting and deconvolution were performed

to improve the interpretation of overlapping diffraction features characteristic of partially ordered and amorphous carbon-based materials.

2. Surface area and porosity analysis (BET)

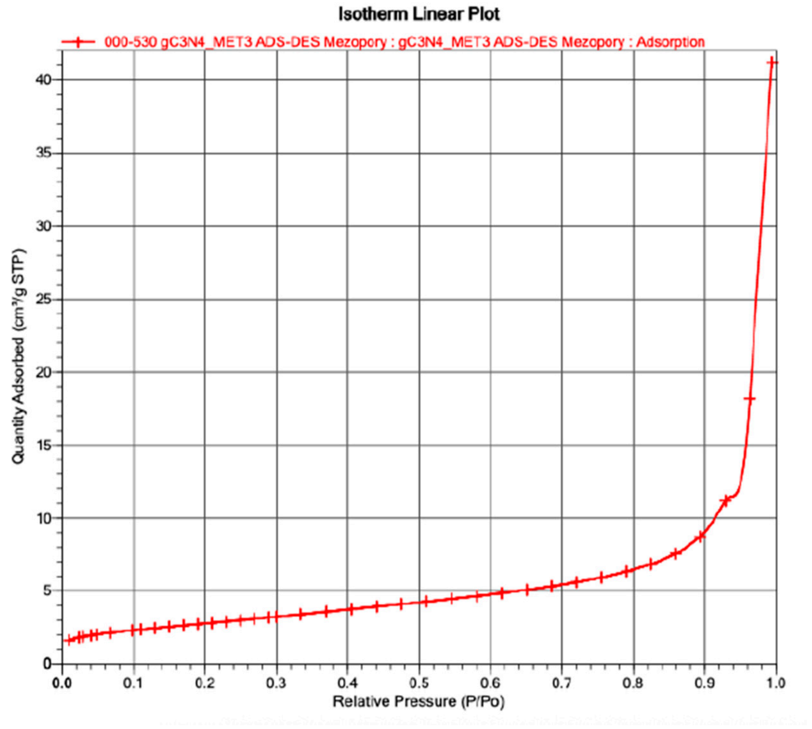

Figure S1 N_2 adsorption–desorption isotherms of pristine g-C_3N_4

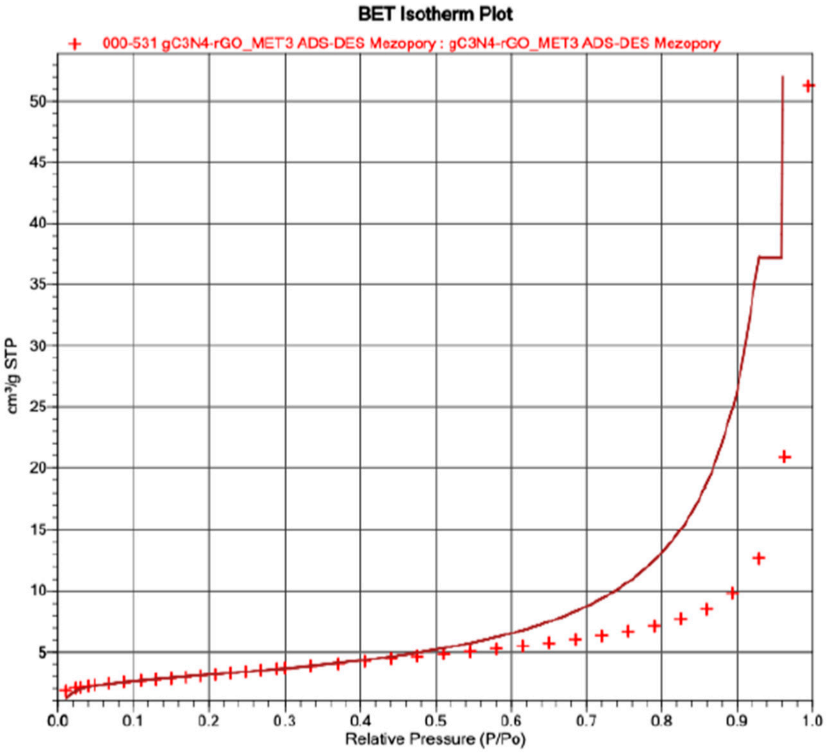

Figure S2 N_2 adsorption–desorption isotherms of the g-C_3N_4/rGO nanocomposite

3. Raman spectroscopy

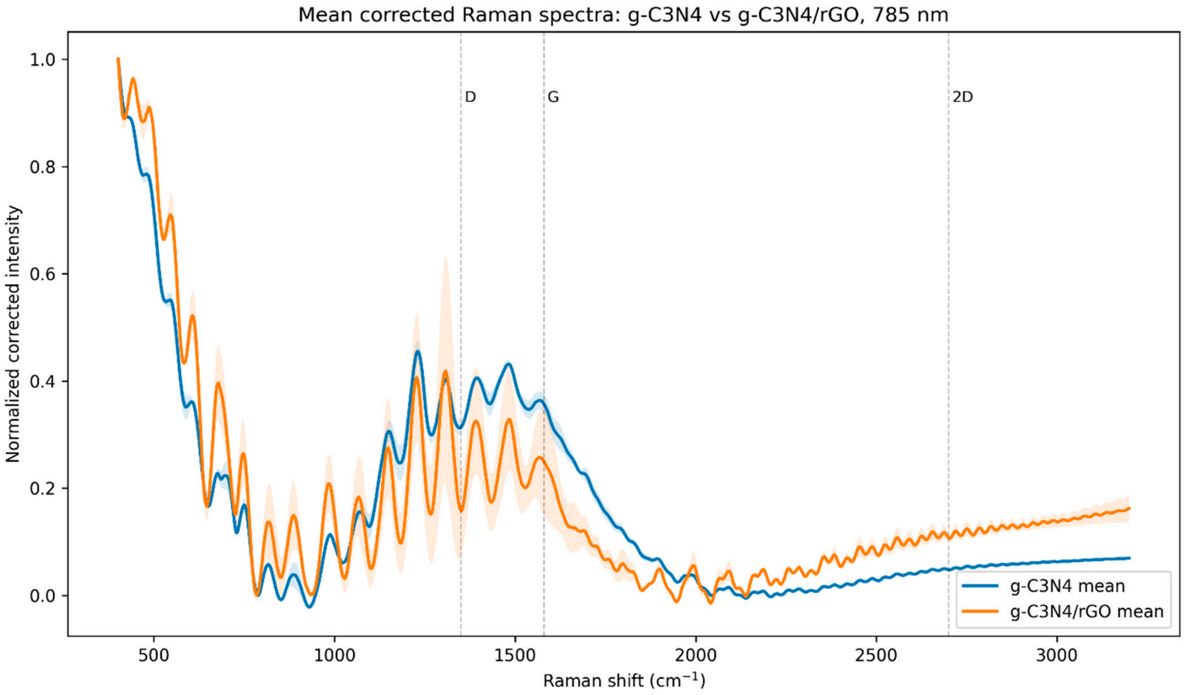

Figure S3 Mean baseline-corrected and normalized Raman spectra of pristine g-C_3N_4 and the g-C_3N_4/rGO nanocomposite recorded using a 785 nm excitation laser. Shaded areas represent standard deviations calculated from two independent measurement points.

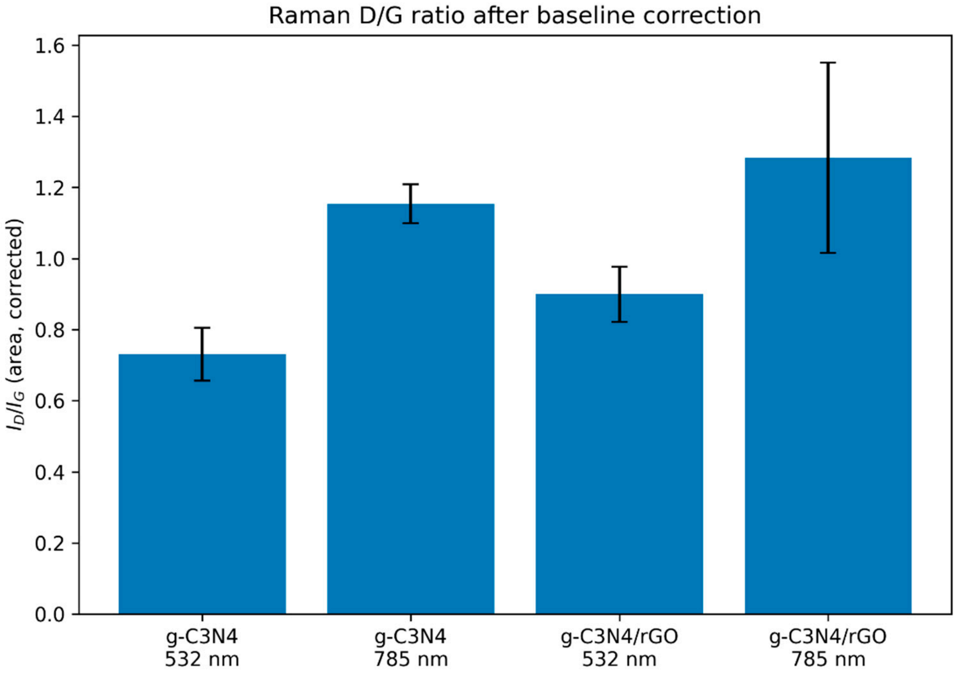

Figure S4 D-to-G band intensity ratios (ID/IG) determined from baseline-corrected Raman spectra recorded at 532 and 785 nm excitation wavelengths for pristine g-C_3N_4 and the g-

C_3N_4/rGO nanocomposite. Error bars represent standard deviations from replicate measurements.

Section S2. Pharmaceutical degradation

Table S1 Raw concentration data obtained during the degradation of pharmaceuticals under UV, UV/O_3, and UV/g-C_3N_4/rGO treatment processes

Process	Time (min)	SMX (mg·L^{-1})	CBZ (mg·L^{-1})	DCF (mg·L^{-1})	IBU (mg·L^{-1})
UV/O_3	0	48.7342	46.0623	44.4620	50.9868
UV/O_3	30	8.3173	15.4929	5.1373	17.3141
UV/O_3	60	6.7795	9.1476	5.0200	9.2566
UV/O_3	90	6.6805	6.6330	n.d.	6.8707
UV/O_3	120	n.d.	5.7759	n.d.	n.d.
UV	0	38.4704	36.1522	34.6646	40.4942
UV	30	11.4480	32.5560	5.0628	32.8713
UV	60	8.1574	30.0526	n.d.	28.7865
UV	90	7.1519	27.4499	n.d.	24.4499
UV	120	6.9723	25.4774	n.d.	23.2415
UV/g-C_3N_4	0	49,0628	46,9139	45,151	52,2014
UV/g-C_3N_4	30	16,3978	40,9703	5,7431	44,6885
UV/g-C_3N_4	60	10,7132	38,6902	n.d.	40,4494
UV/g-C_3N_4	90	8,5346	36,0446	n.d.	36,5817
UV/g-C_3N_4	120	7,6207	34,12	n.d.	35,8025
UV/g-C_3N_4/rGO	0	49.0628	46.9139	45.1510	52.2014
UV/g-C_3N_4/rGO	30	n.d.	35.9050	n.d.	31.7250
UV/g-C_3N_4/rGO	60	n.d.	32.4390	n.d.	29.4160
UV/g-C_3N_4/rGO	90	n.d.	29.6090	n.d.	24.3310
UV/g-C_3N_4/rGO	120	n.d.	26.9060	n.d.	20.1040

n.d. – not detected

Table S2 Pseudo-first-order kinetic parameters calculated for the degradation of pharmaceuticals during UV, UV/O_3, and photocatalytic UV/g-C_3N_4/rGO processes

Process	Compound	n points	k_{obs} [min^{-1}]	SE_k	CI95_low	CI95_high	R^2	$t_{1/2}$ [min]
**UV/O_3**	SMX	4.0000	0.0206	0.0098	-0.0216	0.0627	0.6878	33.72
**UV/O_3**	CBZ	5.0000	0.0167	0.0035	0.0055	0.0279	0.8824	41.58
**UV/O_3**	DIC	3.0000	0.0364	0.0205	-0.2247	0.2974	0.7579	19.07
**UV/O_3**	IBU	4.0000	0.0221	0.0041	0.0044	0.0399	0.9348	31.32
**UV**	SMX	5.0000	0.0130	0.0045	-0.0013	0.0272	0.7370	53.51
**UV**	CBZ	5.0000	0.0029	0.0001	0.0026	0.0032	0.9968	238.88
**UV**	DIC	2.0000	n.d.	-	-	-	-	-
**UV**	IBU	5.0000	0.0047	0.0005	0.0031	0.0063	0.9666	147.85
**UV/g-C_3N_4**	SMX	5.0000	0.0146	0.0037	0.0029	0.0263	0.8392	47.50
**UV/g-C_3N_4**	CBZ	5.0000	0.0026	0.0003	0.0017	0.0035	0.9645	271.84
**UV/g-C_3N_4**	DIC	2.0000	n.d.	-	-	-	-	-
**UV/g-C_3N_4**	IBU	5.0000	0.0032	0.0005	0.0018	0.0046	0.9440	217.89
**UV/g-C3N4/rGO**	SMX	1.0000	n.d.	-	-	-	-	-
**UV/g-C3N4/rGO**	CBZ	5.0000	0.0044	0.0007	0.0022	0.0065	0.9335	159.38
**UV/g-C3N4/rGO**	DIC	1.0000	n.d.	-	-	-	-	-
**UV/g-C3N4/rGO**	IBU	5.0000	0.0073	0.0012	0.0033	0.0112	0.9187	95.66

kobs—apparent pseudo-first-order rate constant; SE_k—standard error of kobs; CI95—95% confidence interval; R^2—coefficient of determination; t1/2—half-life time; n.d.—not determined due to rapid degradation within the initial sampling interval.
